# Commentary: Is the ring the lord of all problems and solutions in mitral valve repair?

**DOI:** 10.1016/j.xjtc.2021.10.037

**Published:** 2021-10-21

**Authors:** Kanika Kalra, Kendra J. Grubb

**Affiliations:** Division of Cardiothoracic Surgery, Emory University, Atlanta, Ga

**Figure:**
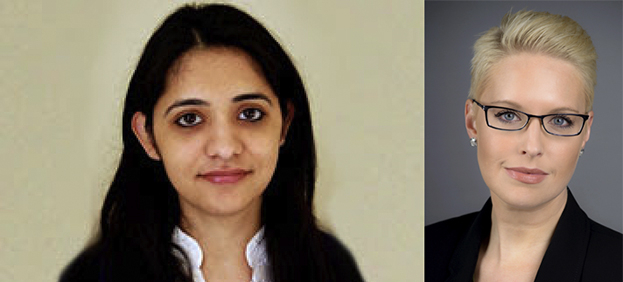
Kanika Kalra, MD, Cardiothoracic Surgery Fellow, Cardiac Track, Emory University, and Kendra J. Grubb, MD, MHA, FACC, Surgical Director, Structural Heart and Valve Center, Emory University.

**Central Message**

The mitral valve repair debate: partial annuloplasty preserves native mitral annular dynamics and facilitates valve closure versus rigid rings fix the annulus at the systolic size aiding coaptation.

See Article page 37.

Mitral annuloplasty to true-size, down-size, or reinforce the native annulus is a mainstay of mitral valve repair for degenerative mitral regurgitation (DMR). In patients with DMR, a certain degree of annular dilatation is observed alongside leaflet or chordal pathology. Restoring the annulus to its true size, reinforcing, and stabilizing achieves appropriate coaptation and proper valve closure. However, success of any annular adjustment depends on the extent of leaflet available and mobility for coaptation. If leaflet and chordal pathology is addressed, and adequate leaflet length and mobility are preserved, then the question of an appropriate annuloplasty prosthesis arises.

Whether to choose a flexible/semi-rigid partial prosthesis or a rigid complete ring has been debated. Those who prefer partial annuloplasty believe that the native mitral annular dynamics are preserved and contribute to valve closure, whereas those who choose a rigid complete ring believe fixing the annulus at the systolic size aids coaptation.^1^, ^2^, ^3^, ^4^ There is merit to both ideas, but consensus is lacking.

In the normal mitral valve, the mitral annulus contracts from its enlarged size in diastole to its contracted state in late diastole to early systole.^5^, ^6^, ^7^ The timing of such contraction is owed to the circumferential fibers from the left atrium that surround the mitral annulus, and the overlying oblique fibers from the left ventricle. Thus, annular contraction coincides with atrial contraction, and continues with left ventricular contraction, twist, and torsion. This rapid contraction draws the mitral leaflets into the mitral orifice for coaptation. By mid to late systole, the annulus enlarges slightly in the anterior–posterior and commissure–commissure dimensions. The leaflets continue to coapt and seal despite mild annular enlargement, as the higher transmitral pressure gradient pushes the leaflets further toward the mitral annular plane.

In this issue of *JTCVS Techniques*, James and colleagues^8^ provide an interesting institutional perspective highlighting the benefits of the semi-rigid posterior annuloplasty band for DMR. As the inventors of the band, they have significant experience and have shown excellent durability with low rates of functional mitral stenosis and systolic anterior motion. The authors owe this to the superior annular dynamics that allows for diastolic relaxation of the annulus and systolic posterior motion of the anterior annulus and the subaortic curtain. They tout a durable, physiologically normal repair, with a degree of flexibility.

Although an enticing hypothesis, for preservation of post-annuloplasty dynamics, pre-annuloplasty native valve dynamics need to exist. When the annulus is significantly dilated, common in DMR or mixed pathology, annular dynamics are not entirely preserved.^9^ Does a semi-rigid partial band allow for dynamic support in this setting? The anterior–posterior dimension does not change from diastole to systole, but the commissure-to-commissure dimension enlarges. Thus, implanting a semi-rigid posterior annuloplasty band can at best follow these dynamics or resist these dynamics to a certain extent, because of its greater stiffness than the tissue. Patients with preserved ventricular contraction, and without any atrial remodeling from mitral regurgitation, would have preserved annular dynamics, which this partial band might help preserve better than a rigid annuloplasty ring.
